# Acceptability and perceived feasibility of the *KaziKidz* health promotion intervention among educators and caregivers in schools from South Africa: a qualitative descriptive study

**DOI:** 10.1186/s12889-024-18456-3

**Published:** 2024-04-01

**Authors:** Patricia Arnaiz, Manfred Max Bergman, Harald Seelig, Larissa Adams, Danielle Dolley, Markus Gerber, Nandi Joubert, Siphesihle Nqweniso, Peter Steinmann, Jürg Utzinger, Rosa du Randt, Cheryl Walter, Uwe Pühse, Ivan Müller

**Affiliations:** 1 Department of Sport, Exercise and Health, University of Basel, Basel, Switzerland; 2 Department of Social Sciences, University of Basel, Basel, Switzerland; 3 Department of Human Movement Science, Nelson Mandela University, Gqeberha, South Africa; 4 Swiss Tropical and Public Health Institute, Allschwil, Switzerland; 5 University of Basel, Basel, Switzerland

**Keywords:** School-based interventions, Physical activity, Health promotion, Acceptability, Feasibility, Teacher, Caregiver, South Africa

## Abstract

**Background:**

Despite the uncontested benefits of physical activity, its promotion lags behind in the public health agenda of low- and middle-income countries (LMICs). School-based interventions are promising strategies to foster health during childhood, but evidence of their effectiveness is limited and inconclusive for LMICs. Thus, further investigation is needed on contextual factors associated with intervention implementation in low-resource settings. We studied the acceptability and feasibility of the *KaziKidz* health promotion intervention and its implementation and make recommendations to improve future adoption and sustainability.

**Methods:**

*KaziKidz* was implemented in four primary schools from low-income communities in South Africa in 2019. Semi-structured interviews with four school principals, three focus group interviews with 16 educators, and another three with 16 caregivers were conducted between October and November 2021. Participants were purposively recruited. Interview transcripts were analyzed via thematic analysis using a deductive and reflexive approach.

**Results:**

Three main themes influencing intervention implementation and adoption were identified: (1) prioritizing teachers’ needs (2), integrating the program into the school structure, and (3) creating opportunities in the community. Supporting recommendations included: (theme 1) adopting intervention approaches that are inclusive of educators’ health and providing them with capacity development and external support; (theme 2) fostering a feeling of ownership and belonging among school stakeholders to adapt interventions to specific resources and needs; and (theme 3) raising community awareness to encourage individuals to claim power over and actively engage with the program.

**Conclusions:**

Comprehensive interventions comprising health support, adequate training, and ongoing assistance for educators combined with school-wide and community outreach actions seeking to strengthen program ownership, accountability, and engagement can enhance uptake of school-based interventions and long-term maintenance.

**Trial registration:**

ISRCTN15648510, registered on 17/04/2020.

**Supplementary Information:**

The online version contains supplementary material available at 10.1186/s12889-024-18456-3.

## Introduction

The benefits of physical activity (PA) for health and adequate development in childhood are widely recognised [1–3]. Yet, physical inactivity is widespread among school-aged children. In Sub-Saharan Africa, the prevalence of insufficient PA among adolescents amounts to 86%, making it the second-highest globally [4]. Despite physical inactivity being long identified as a global health issue and a pandemic in itself [5], the promotion of PA has received little attention, particularly in low- and middle-income countries (LMICs), where infectious disease prevention remains the primary focus of the public health agenda [6]. As a result, little progress has been observed in promoting PA in LMICs [7]. The Global Observatory for Physical Activity reports high inequalities among countries and regions in their capacity for promoting PA, with Africa having the lowest capacity [6]. Therefore, there is a call to action to implement effective policies and programs to increase PA levels in the African population, especially in children [8].

School-based interventions are promising and cost-effective strategies to foster healthy, active lifestyles early in life [9]. The multi-component *KaziKidz* program was developed based on the UNESCO criteria for Quality Physical Education and its “Fit for Life” project to promote health and physical literacy in under-resourced public primary schools in South Africa (SA) [10, 11]. Positive intervention effects on cardiovascular parameters and PA levels have been observed for the *KaziKidz* program [12, 13] and similar comprehensive interventions [14]. Still, inconsistent results on the effectiveness of school-based health-enhancing interventions have been reported worldwide [15] and in Africa [16]. A systematic review found differences between rural and urban settings [17], suggesting that the context influences program success. The discrepant results and limited evidence from LMICs [18] underscore the need to investigate contextual factors and specific challenges associated with program implementation in low-resourced schools.

Implementation research seeks to facilitate the uptake of evidence-based practices in real-life settings [19]. It aims to ensure effective implementation and sustainment of interventions by identifying and addressing context-specific challenges that influence their delivery. To examine the conditions surrounding the implementation of interventions, Proctor and colleagues defined a series of implementation outcomes [20]. Outcomes such as acceptability, i.e. the perception among implementation stakeholders, and feasibility, i.e. the extent to which an intervention can be successfully implemented, are crucial to recognizing areas of weakness. While acceptability and feasibility are typically assessed during the early stages of a program, conducting a post hoc evaluation of these outcomes can offer valuable insights into the implementation process and serve as explanation for the program’s success or failure [20, 21].

Indeed, poor deployment can hinder the success of otherwise effective initiatives. The importance of assessing the implementation quality of school-based health interventions has been previously reported [22]. Authors have claimed that strengthening implementation research on PA programs can provide context-specific information that supports schools in their effective implementation [23]. While several studies have described facilitators and barriers of school-based PA interventions, evidence suggests that further research is necessary to develop strategies that put this evidence into action [24]. Thus, a need exists to accelerate our understanding of successful implementation strategies and ensure that effective interventions are successfully established in diverse settings and populations.

Hence, the main purpose of this paper is to inform practitioners and policy makers on evidence-based implementation strategies to guide the future of *KaziKidz* and other health promotion interventions in schools. Specifically, we aimed to understand the acceptability of the *KaziKidz* intervention by exploring educators’ and caregivers’ perceptions thereof, and to analyze the feasibility of both the intervention and its delivery by learning about the schools’ experiences with the program implementation. Finally, we identify points of action and make specific recommendations to improve the adoption, sustainability, and ultimately effectiveness of school-based interventions.

## Methods

### Study design

This qualitative descriptive study is part of a mixed-methods project evaluating two school-based interventions: *KaziKidz*, a health promotion program for learners, and *KaziHealth*, a workplace health intervention for educators [25]. Both interventions were implemented in the context of a randomized controlled trial in eight participating schools, four intervention and four control, in 2019 [10]. Thereafter, the follow-up evaluation study involved a series of quantitative and qualitative assessments in 2021, using a convergent design to collate and analyze these data separately and draw overall conclusions [26]. This paper examines the qualitative outcomes of the *KaziKidz* intervention and presents them in a descriptive manner.

### Intervention

**Fig. 1:**
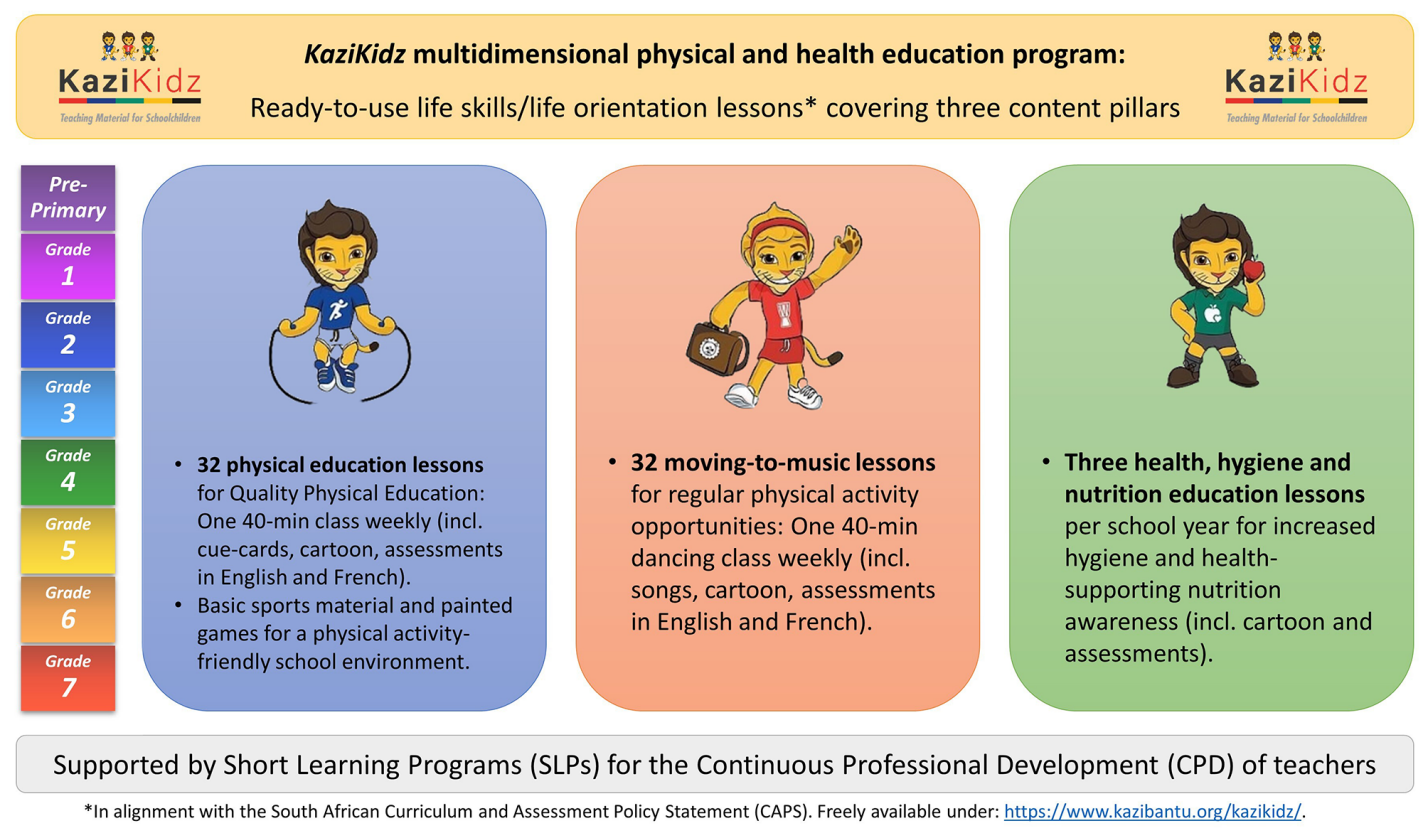
The *KaziKidz* intervention framework encompasses context-adapted lessons, environmental modifications, and teacher support strategies, aiming to promote holistic education and well-being among students from pre-primary to grade 7

In the South African school system, physical education (PE) forms part of the life skills/life orientation (LS/LO) subject. Generally, PE is taught by non-specialists and little time is dedicated to it, approximately one hour per week per grade [27]. The *KaziKidz* program aims to support LS/LO educators in delivering quality and comprehensive physical and health education. Its multi-component approach includes context-adapted and ready-to-use teaching materials with PA and dancing exercises, as well as health, hygiene, and nutrition lessons for grades 1 to 7; teacher training in the form of workshops or direct support from trained coaches; and environmental changes, like the provision of basic PA equipment (balls, ropes…) and games painted in the playground (hopscotch, four-square…) [28]. A graphical intervention outline is provided in Fig. 1.

*KaziKidz* is coupled with the *KaziHealth* workplace intervention, which seeks to promote healthy lifestyles among primary school educators. *KaziHealth* consists of an individualized health risk assessment followed by lifestyle coaching that aims to increase personal knowledge and motivation.

### Study setting

The participating schools were located in peri-urban, low-income areas in the Nelson Mandela Bay Municipality of the Eastern Cape Province of SA. These areas represent historically disadvantaged communities, enduring spatial, social, economic, and political marginalization. Despite their close proximity to more urban, developed, affluent centers, these communities continue to grapple with persistent challenges such as poverty, inadequate housing, high unemployment rates, and elevated crime levels [29].

All eight schools are classified as public primary quintile 3 schools on the national poverty scale, where 1 represents the poorest and 5 the least poor schools, and are non-fee-paying institutions. The historical legacy of inequality in schools situated in lower-income settings prevails in the unequal availability and condition of sports equipment and facilities [30]. The effective delivery of physical education in these schools is further hindered by high teacher workload, large class sizes, and insufficient funds. Additionally, limited opportunities for physical activity stem from the absence of, or inaccessibility to, organized sports, activity clubs, or chances for outdoor play [31].

Children from the *KaziKidz* intervention, aged 8 to 13 years old, had a median socioeconomic status of 6, as determined by a nine-item questionnaire, where 0 represents the lowest housing characteristics and household possessions and 9 the highest [13]. Notably, children exhibited an unfavorable cardiovascular profile, with 56% manifesting at least one risk factor for non-communicable diseases (NCDs). Elevated blood pressure, overweight, and physical inactivity were the most prevalent among them. Specifically, more than one-third (36%) of children failed to meet the recommended daily activity levels of 60 min of moderate-to-vigorous intensity physical activity.

### Participants

Purposive sampling was employed to identify study participants in the schools that implemented *KaziKidz* in 2019. School principals from the four intervention schools were invited to take part in the interviews. Three of these schools were asked to recruit eight educators and eight caregivers each to reach a total sample of 48 participants for the focus group interviews (FGIs). The final study sample consisted of four school principals, two women and two men, 16 educators, 12 women and 4 men, and 16 caregivers, 14 women and 2 men.

### Data collection

Four semi-structured interviews with school principals and six FGIs, three with educators and three with caregivers, were conducted between October and November 2021. Three different interview guides were developed to reflect the particular role of each group. All guides contained questions pertaining to the participants’ perception of and experiences with the program and its implementation (Additional file 1). Furthermore, prompts and follow-up questions were employed to invite interviewees to elaborate on their responses and maintain a balanced participation during the FGIs. Interview guides were pilot tested, and questions adapted accordingly.

All interviews and FGIs were conducted in-person, at the schools, and in English by an independent researcher, who was familiar with the research setting and briefed on the study. The first author (PA) was present in all interviews acting as observer and note-taker. A Xhosa native speaker, who is part of the research team, was also present and assisted with translation whenever needed. All sessions were audio recorded and lasted an average of 24 min for interviews with school principals and an average of 60 and 43 min for FGIs with educators and caregivers, respectively.

### Data analysis

The interviews were transcribed verbatim and analysed via thematic analysis following the six-phase model proposed by Braun et al. in an iterative and reflexive manner [32]. The analysis was done by the first author (PA), a woman researcher with a public health background, who approached the analysis in a primarily semantic and inductive manner. The six analysis steps included (i) familiarization with the data, which started by taking notes during the interviews and continued by transcribing and re-reading the transcriptions; (ii) initial open coding in MAXQDA Plus 2022 (VERBI Software, Berlin, Germany), followed by a revision that led up to more latent and nuanced codes; (iii) theme development, which involved the arrangement of codes into preliminary themes informed by thematic frameworks from the literature; (iv) theme refinement and (v) naming entailed a detailed analysis of the data with the help of thematic maps to find meaning across the dataset and alignment with the research question, which led to new, crosscutting themes; and (vi) writing up the report by collecting, editing, and situating the analysis in context. During the entire analytical process, PA collaborated closely with senior authors MB, a social science researcher, and HS, an experienced methodologist, who reviewed the coding, agreed on the analytical framework, and participated in theme refinement. Compliance with Braun et al.’s 15 criteria for good thematic analysis ensures methodological quality and rigor of the analysis (Additional file 2) [33].

## Results

Data from school principals, educators, and caregivers were combined to present a contextualized and in-depth picture of the *KaziKidz* program implementation in four primary schools in SA. Three main thematic areas were developed across the interviews and FGIs relating to the participants’ perceptions of the program and experiences with its implementation.

Table 1 presents the three key themes and sub-themes generated through the thematic analysis. The main themes were: prioritizing teachers’ needs, integrating the program into the school structure, and creating opportunities in the community.

**Table 1:** Main themes and sub‑themes arising from transcript analysis

Main themes	Sub-themes
1. Prioritizing educators’ needs	1.1. Health and lifestyle
	1.2. Workload and expertise
2. Integrating the program into the school structure	2.1. Stakeholders’ role
	2.2. Curriculum configuration
	2.3. Availability of resources
3. Creating opportunities in the community	3.1. Child development
	3.2. Caregiver involvement

### Theme 1: prioritizing educators’ needs

School personnel expressed seeing educators as pivotal figures in program implementation and hence, their buy-in necessary for program success. However, a number of issues related to their personal health and professional responsibilities that influenced their motivation and availability to engage with the program arose in their discourse.

#### 1.1 Health and lifestyle

Despite the fact that no questions concerning educators’ health state were included in the interview guide, several participants mentioned health conditions and associated them with unhealthy lifestyles, for example sedentary behavior:

*“I have arthritis, I’m truly lazy, sometimes when I come to school I request an Uber going home because it’s hard to walk.” (Program coordinator, woman, school 3)*.

In turn, unhealthy habits and unfavorable attitudes toward sport were reported to have a negative impact on how educators engaged with the program.

*“After the [KaziHealth health assessment] I discovered that I’m unhealthy with certain areas. I tried to be conscious, but to bring it down to the kids is not that easy. So I do not encourage my learners. The fact is… activity to me is strenuous and I’d rather sleep than exercise.” (Educator, woman, school 1)*.

Meanwhile, educators from one school described being physically active and interested in sports as a sign of predisposition to get involved with the program.

*“Many of us here are physically busy, exercising and so, so we will definitely get on board with this thing.” (Educator, man, school 1)*.

Educators’ mental health seemed to be an issue of concern too.

*“As we come to work each day we have a lot of challenges…We need moments where our cup is filled again, talk about our mental health, because if we’re not mentally healthy, our bodies are going to feel weaker.” (School principal, woman, school 3)*.

#### 1.2. Workload and expertise

A high workload and pressure from the Department of Education to finalize the school curriculum was consistently reported. According to school principals and educators, the multitude of duties that educators must undertake on a daily basis engenders a sense of stress and a dearth of available time.

*“There’s not really time for them [educators]… the pressure from the department to finish the curriculum and complete the FATs, the formal assessment tasks, puts lots of pressure on the teacher.” (School principal, man, school 2)*.

The stated pressure and scarcity of time undermined educators’ motivation to engage in non-academic and extracurricular activities.

*“The problem is time… We don’t have the time because we are pushed, we have our own academics and things that we need to finish.” (Educator, man, school 1)*.

Moreover, educators’ inadequate training in PE was raised in accordance with the program’s potential to increase teaching competency, which was regarded as positive.

*“Programs like this just enrich and give us more opportunities, more grounds to speak on.” (Educator, man, school 1)*.

Therefore, the educators’ disposition toward the program was positive and hinted at a willingness to overcome the cited barriers and engage with the program:

*“Since it’s something that is for fitness, for health… I think it’s very important. Because sometimes we get tired of even reading things, just taking the forms that you have given us, we’ll say ’nah nah, we have to read now again?’ But for the things that are important, we can do it.” (Educator, woman, school 1)*.

### Theme 2: integrating the program into the school structure

Embedding the program into the school day-to-day operations was also widely recognized to be key for successful program implementation and maintenance. It seemed that having a clear structure would minimize the stated motivational and logistical barriers. Nonetheless, respondents reported that adopting the intervention required a systemic approach, whereby diverse aspects of the school environment needed to be considered.

#### 2.1. Stakeholders’ role

It was outlined that establishing a school-wide initiative requires an effective and joint effort from all stakeholders involved and not just from educators.

*“You need the SGB [School Governing Body], Department of Education, teachers, kids, everyone to buy into this idea… because it’s a wonderful initiative, but it has to have everybody’s involvement to get this program running and to operate at the optimal level.” (Educator, man, school 1)*.

On the one hand, school principals acknowledged their role as a motivating force in launching the program and stimulating enthusiasm among educators.

*“The school has got to come together and look at this thing in depth… It’s got to start here in the office with a plan, and I’m sure by doing that the teachers will get on board.” (School principal, man, school 1)*.

On the other hand, educators articulated that their active participation was imperative not only for program implementation but also to instill motivation in children. The perception of themselves as role models created a sense of responsibility that fostered their engagement.

*“If I was just standing there, just being there, the learners will definitely not be interested. So I must show interest for them to be interested.” (Educator, woman, school 2)*.

Further, other influential stakeholders were mentioned. Specifically, the Department of Education was alluded to in its capacity as a policymaker and its potential to support incorporation, establishment, and dissemination of the program.

*“The Kazi project is a good thing, but you need to bring it more to the front, speak to the people that’s there in charge.” (Educator, man, school 1)*.

#### 2.2. Curriculum configuration

A prevailing agreement was observed regarding the syllabus’ pronounced focus on academic performance, which together with the cited tight schedule and low teaching proficiency and motivation, contributed to the neglect of PE.

*“So there is a program out but with limited emphasis on physical education… Learners learn through play and being active, but they don’t get that opportunity. It’s more academics in the classroom.” (Educator, man, school 1)*.

Caregivers reinforced the perceived superiority of academic subjects by asserting that the amount of time allotted to PA decreases as children mature and require greater concentration on academic pursuits.

*“Ever since grade five, in the afternoon, from half past three to nine o’clock at night we will sit with homework. There’s no time for after school curriculums… I had to cut him off because his school marks came down a lot, so he had to focus more on school.” (Caregiver, man, school 1)*.

Nevertheless, principals, educators, and caregivers alike agreed on the importance of adopting a holistic approach to education. They affirmed that the program’s worth resided in its potential to complement the existing syllabus with non-academic content.

*“It took the curriculum to another level in terms of showing the children besides the book work. We do a lot of book work… Where Kazi came and it showed the children, ‘enjoy what you’re doing’.” (Educator, woman, school 2)*.

Finally, educators recognized that the *KaziKidz* program aligned well with the current curriculum, reinforcing existing topics and providing practical teaching resources and tools.

*“It does fit in the school curriculum because… everything that is taught on the life skills is what they are doing in Kazi, so that it helps them even more on that [teaching LS/LO].” (Educator, woman, school 3)*.

#### 2.3. Availability of resources

Several respondents mentioned that the schools’ poor infrastructure and inadequate sports equipment were hindering factors for the practice and teaching of PE.

*“Tools can also motivate the teachers to be able to take the children for physical education, but we don’t really do it properly because of the lack [of equipment].” (School principal, woman, school 4)*.

The resources offered with the program, namely, simple PA equipment, painted games, and posters, were consistently reported to assist teaching.

*“Because they [educators] were also given certain tools and materials, which the school did not have… the teachers were able to take the children to the court… So I think that part also assisted a lot the educators.” (School principal, woman, school 4)*.

The materials provided also encouraged movement among learners.

*“Those things [skipping ropes] even they [younger children] use it, they enjoy those types of things… the poorer schools that we are in don’t have those resources” (Educator, woman, school 2).*

Resources other than infrastructure were mentioned by principals and educators. Concretely, staff shortage and teacher turnover indicated a negative impact on program maintenance.

*“Another principal told me that three people [are] sick. The principal is also out of school more than a year now… And there is no money to pay substitutes.” (School principal, man, school 2)*.

*“Mrs [name] used to run the program, and then… she passed away, so she was the one that had control over all the facets of the program.” (Educator, man, school 1)*.

### Theme 3: creating opportunities in the community

The perceived value of the program for children and its potential to have a positive impact on the wider community was identified as a significant factor contributing to its acceptability. This was especially true in connection with the unfavorable circumstances that characterize the study setting, such as poverty, unemployment, or crime, and hence, the opportunities that providing a holistic, quality education could bring.

#### 3.1. Child development

Educators and caregivers noted that *KaziKidz* had a positive effect on children’s wellbeing, behavior, and discipline.

*“You can even see after the program the children got more self-confidence… They have a more positive mindset now.” (Caregiver, woman, school 2)*.

Moreover, it was mentioned that lifestyle programs like the *KaziKidz* promoted tolerance and cooperation among peers, attitudes that are especially important in diverse communities.

*“They learn team work. Most people come [to school] with different backgrounds, so they have to be tolerant of one another… So it is important that they get into those programs.” (School principal, woman, school 3)*.

Participants also highlighted the significance of such programs for diversity and inclusion, as they provide opportunities for children who struggle with academic subjects and serve as motivators for attending school.

*“They were quite excited to come to school. Some of the learners don’t learn in class because of their own barriers. But when it comes to coming out and play, they enjoy that. So they come to school just for the sport and the physical education.” (Educator, woman, school 2)*.

Overall, the program’s potential to cultivate children’s mindset, discipline, tolerance, and inclusion resulted in a sense of opportunity that is often absent in these communities.

*“[The] KaziKidz program it’s quite a good program for our school, taking into account the community that our learners are coming from, where unemployment rate is high, crime, poverty… We should be making sure that [each] child gets proper education and is equipped for the future.” (School principal, woman, school 3)*.

#### 3.2. Caregiver involvement

Accounts from principals and educators reflected a strong wish to involve children’s guardians in the program, with the goal of adopting the knowledge and habits beyond the school setting and ultimately impacting the broader community.

*“Who is the driving engine of the kids after school? It’s the parents… They are stakeholders of it… because you are only stuck with the kids for certain amount of hours.” (Educator, man, school 1)*.

Caregivers themselves reported that information had gotten through to the home setting.

*“Kids sometimes tell us… “don’t eat too much food, don’t eat too much meat’. When there are programs here in school, kids learn fast, then they come to us and they teach us.” (Caregiver, woman, school 3)*.

However, their narrations demonstrated limited understanding. While some guardians mentioned not receiving any information, those who were aware of the program often confused it with the data assessment.

*“I don’t actually have a lot of information about this… Just my child was participating in it and she brought the thing [accelerometer] that she had to put on the stomach.” (Caregiver, woman, school 2)*.

Moreover, respondents recognized that adopting healthy lifestyles is challenging given the adverse conditions of the communities they live in.

*“There is a lot of unemployment in this community, so even to change your diet some of us can’t afford it… Normally we do food that goes a long way.” (Caregiver, woman, school 2)*.

In general, caregivers expressed a desire to be more informed about the program and to support their children.

*“Bring the parents also to the table, so they can have a better understanding, we will know better how to answer all the questions [from children regarding the program].” (Caregiver, woman, school 2)*.

## Discussion

Participants provided insight into their perceptions and experiences for implementing the *KaziKidz* program in public primary schools. School principals and educators’ narrations revealed that attending to educators’ personal and professional needs is central to strengthening their engagement with the program. Furthermore, integrating the program into the school system was recognized to facilitate delivery and maintenance. Finally, respondents acknowledged that adoption beyond the school setting is necessary to ultimately create a positive impact in the community.

The first theme identified in our study places educators in the spotlight of school-based interventions. We found that poor health and unhealthy lifestyles conditioned their attitude toward sport and ultimately hindered their engagement with the intervention. Notwithstanding the dependability on educators for implementing school-based programs, there has been a failure to acknowledge the influence of their health and wellbeing in adopting and sustaining said programs. While efforts to improve teachers’ health exist, these have been essentially done in isolation as opposed to being part of dual-approach programs tackling learners and educators’ health simultaneously [34]. The encountered conditioned narrative around educators’ health is in line with a study from a similar context in SA [35], where the authors concluded that educators should be included as recipients of health-promoting programs carried out in schools. Yet, a review from 2021 shows that interventions targeting teachers’ wellbeing are still scarce and more research is required [36]. Furthermore, the suggested increased acceptability following educators’ own intervention participation aligns with previous research showing that training concentrating on the wellbeing of teachers significantly increased their intention to implement school-based programs [37]. Thus, in addition to the personal benefits for teachers themselves, attending to teachers’ health and wellbeing can have a positive impact on the success of interventions. Nevertheless, other issues pertaining to the professional level were stated to have impacted their engagement with the program.

Our findings on barriers to implementing school-based PA programs are consistent with those frequently described in the literature, which include high workload, pressure to finalize the curriculum, and reduced time dedicated to non-academic subjects [21, 38, 39]. The limited subject knowledge described in this study has also been reported as a hindering factor to delivering PE, together with the need for continued professional development (CPD) to increase subject competence and teaching confidence [40]. Indeed, previous research has shown a good acceptance of teacher training designed to incorporate PA into the classroom as well as an improved teaching efficacy [21, 41]. Strategies to increase PE teaching competencies become particularly relevant in the South African context, given the limited training that LS/LO teachers receive during their professional education. Stroebel and colleagues confirmed that a major challenge for delivering PE in South African schools was the absence of qualified PE teachers. Hence, they advocate for collaboration between authorities and universities to offer adequate PE-specific in-service training for LS/LO teachers [42]. Given the previously exposed barriers, it is imperative to establish schemes that provide ongoing support to teachers both in a personal and professional capacity.

The second theme generated through our analysis shifts the focus from the educators to the school. The consensus that school accountability on academic performance pushes PE and extracurricular activities to the background was coupled with a positive view of the program’s holistic educational approach. Indeed, the multi-component nature of the *KaziKidz* toolkit that integrates PA promotion, nutrition, and general education for healthier lifestyles has been described as a characteristic of successful intervention programs [43]. Moreover, whole-school approaches are long recognized as a requirement for “Health Promoting Schools” [44], whereby different strategies are put in place to promote health beyond curricular education. The *KaziKidz* intervention builds on recommendations for school-based health promotion interventions in LMICs and reinforces individual behavioral change activities with improvements in the structural environment [45]. One example is the provision of sports equipment and painted games. As reported by previous studies [46], we found that supplying PA materials was valued, facilitated implementation by overcoming a limitation of resources, and promoted motivation and movement among children. Yet, it was emphasized that a supportive school environment is needed to bring non-academic activities to the foreground and ensure the successful and sustainable implementation of the program. Importantly, the lack of executive support to integrate interventions within the school has been highlighted before as a key obstacle to program sustainment [47]. Therefore, both staff engagement and administrative involvement are key factors in adapting one-size-fits-all approaches to local school health promotion efforts.

Because schools have different agendas, priorities, needs, and values, one-size-fits-all approaches to school-based health interventions might limit adoption. One study from the United Kingdom aimed to increase the autonomy of teachers and found that presenting them with a range of activities to choose from enhanced program adoption [48]. However, the reception of increased self-determination might differ between high-income countries and LMICs. During the implementation of the HealthKick program in SA, the authors observed that teachers appreciated a structured way of preparing and teaching classes [49]. Thus, a better approach to enhance program success in low-resourced schools in SA might be gathering stakeholders’ input on how ready-to-use lessons can be implemented within the school schedule [45].

The last theme of this study concentrates on the importance of bringing new opportunities to deprived communities, especially for children. Caregivers and educators valued the potential of both PA and *KaziKidz* to positively influence children’s physical and psychosocial health and wellbeing. Previous studies have suggested that school health programs can reduce health inequalities in socioeconomically disadvantaged communities, as PA interventions showed more beneficial results among children from low-income households [50]. The program’s capacity to impact the development and social skills of learners was also emphasized. Concretely, better behavior and discipline, as well as the possibility to promote the inclusion of intellectually challenged children were cited. These results align with one study that reported good acceptability and improvement in social-emotional skills of an intervention addressing fundamental motor skills and social and emotional development [51]. However, for the reported benefits to be sustained over time and translated into long-term opportunities, learned behaviors need to be supported by the community. In this regard, a proactive involvement of caregivers could enhance the intervention’s holistic approach, thereby amplifying its benefits. Nonetheless, obstacles to ensuring parental involvement in school-based physical activity interventions persist [52], especially in resource-limited settings [53], raising doubts about whether caregivers’ expressed interest would translate into tangible action.

### Recommendations

Based on direct statements from the participants and identified underlying narratives, we have compiled strategies aiming at increasing the acceptability, feasibility, adoption, and maintenance of school-based health promotion interventions. Designing interventions that are inclusive of educators’ wellbeing and providing them with capacity development could best address personal and professional barriers to program engagement. Furthermore, creating a sense of ownership among stakeholders could motivate the adaptation of the program to the schools’ needs, thereby facilitating its integration. Similarly, involving learners and caregivers through increased awareness could inspire change in the community. Box 1 describes the recommendations per thematic area and we discuss them in the following paragraphs.

**Box 1:** Recommended strategies to increase uptake and maintenance of health promotion interventions in schools

**Recommendations based on theme 1: Prioritizing educators’ health**	
Intervention approach participatory and inclusive of educators’ health	School-based interventions aiming to improve learners’ health should adopt a comprehensive approach, whereby part of the program is specifically designed to attend to educators’ health and wellbeing. A participatory action process should be utilized to co-design the intervention together with educators.
External support and capacity development	An appropriate introduction to the intervention through workshops and demonstration of activities should be ensured prior to implementation. In addition, strategies for capacity development, such as in-service training or CPD credits, should be offered in collaboration with higher education institutions.
**Recommendations based on theme 2: Integrating the program into the school structure**	
Feeling of ownership and belonging	Project partners should focus on transmitting a sense of ownership and belonging to the schools through close collaboration and adequate communication. To further reinforce program ownership by schools, a committee or champion responsible for the program and its implementation should be organized.
Program adaptation to resources and needs	To best embed interventions into schools and avoid delivery barriers, school stakeholders should first reflect on the characteristics of intervention and their available resources and needs. Then, both the intervention and its implementation should be adapted to fit the school requisites.
**Recommendation based on theme 3: Creating opportunity in the community**	
Awareness raising and power claiming	Future interventions should put in place effective communication strategies aimed at raising awareness in the community. A better understanding of the intervention values and goals could encourage caregivers and learners to claim power over the intervention and to adopt its message at home.

### Theme 1: Prioritizing educators’ needs

#### 1. Intervention approach participatory and inclusive of educators’ health

School principals and educators’ stories were closely connected to their own participation in the *KaziHealth* teachers’ health intervention, demonstrating a desire to position their experiences at the forefront of the conversation. In our experience, considering and addressing the individual needs of teachers substantially increased the acceptability of the *KaziKidz* intervention. Thus, we recommend school-based health programs to adopt a comprehensive approach that broadens the program focus from a learner-centered to a school-community perspective, prioritizing the health and wellbeing of both learners and teachers. A recent systematic review of the mental health and wellbeing of school teachers has highlighted the relevance of co-designing health-promoting programs with teachers for teachers [54]. To achieve this, it is crucial to include qualitative exploration of educators’ lived experiences and desires, and to adopt a participatory approach to co-create inclusive interventions [55]. A participatory action process should seek to encourage teachers to take accountability, determining their own priorities and aligning the programme accordingly.

#### 2. External support and capacity development

The type of support received from the research team was found to influence school principals’ and educators’ attitudes toward the intervention and experience with its delivery. Respondents from the schools that, besides the teaching materials, received either no external support or had two workshops, reported a limited understanding of the program. In contrast, the school that had additional support by a coach declared a more positive experience. Nevertheless, shortcomings regarding implementation facilitation were pointed out across all schools. Beyond good acceptability among teachers, it has been suggested that a supportive environment is critical to provide quality PE effectively [21]. Thus, our recommendation to provide external support to kick-start the program together with continuous capacity development is in line with suggestions from Hill and colleagues, which seek to ensure that school-based health interventions are implemented as intended and sustained over time [49].

### Theme 2: integrating the program into the school structure

#### 3. Feeling of ownership and belonging

Participants conveyed an interest in the successful implementation of the program, but frequently attributed the responsibility to external parties. A subset of educators from the unsupported school reported feeling detached from the program due to a perceived insufficient communication regarding their role and common goals. This indicates that close support from external parties can foster a feeling of belonging that motivates educators’ active engagement. Moreover, it is equally important that schools take ownership of the program to ensure meaningful and sustainable implementation. The implied delegation of power suggests a need for greater emphasis on program ownership by schools. In line with earlier studies and participants’ proposals, we advise for a person, champion, or a group, committee, within the schools to be appointed responsible for program coordination [38].Still, the external support outlined in Recommendation 2 should be consistently provided from the initial stages through the sustainability phase.

#### 4. Program adaptation to resources and needs

Our findings suggest that tailoring the intervention and its implementation to the unique characteristics of each school could assist with program delivery and fidelity, and ultimately improve effectiveness. For instance, one recurrent proposal was to allocate a specific time period within the school schedule for *KaziKidz*; however, suggestions on how to incorporate such periods varied between respondents. Therefore, initial collaboration with the schools is essential to ensure that the program addresses real needs and its implementation is feasible. Nevertheless, institutional support is still necessary for further adoption and maintenance. As stated by Lambrinou and colleagues “intervention programs delivered in vulnerable groups and/or low-socioeconomic areas should focus on school policy and environmental changes” (page 17) [56]. Hence, policy frameworks that address curriculum prioritization are needed to facilitate structural changes that allow for health-promoting activities.

### Theme 3: creating opportunities in the community

#### 5. Awareness raising and power claiming

Participants’ accounts suggest that having a clear understanding of the program values and goals is essential not only for educators, but also to foster acceptance and adoption by learners and caregivers. Educators believed that effective communication could raise awareness among guardians and encourage their engagement with the program. Indeed, previous research has shown that involving students actively and giving them greater autonomy to customize interventions creates a sense of accountability and program ownership [45, 48]. Similarly, a systematic review found that activities that sought to engage caregivers directly, such as educational meetings, were more effective than indirect methods [57]. These findings are in line with the educators’ recommendations to actively involve caregivers in the program both to encourage pupil participation and to translate the results to the home context.

### Limitations

The findings of this study provide valuable insights into the experiences of schools located in low socioeconomic communities in the Nelson Mandela Bay Municipality. However, it is noteworthy that the process evaluation was not conducted during the intervention implementation phase due to limited resources and the desire to avoid power dynamics. To address this gap, qualitative examinations were conducted post hoc, shedding light on the actual willingness and capacity of schools to deploy the intervention as intended. Additionally, the lack of implementation monitoring also means that it is not possible to determine if some schools were more engaged with the program or if there were differences in the frequency or delivery mode between schools. This limitation highlights the need for further research into the relationship between intervention acceptability and delivery in multiple settings. Furthermore, despite following standard data collection and analysis procedures to mitigate bias, it is important to acknowledge that complete elimination of bias is impossible, and potential shortcomings still exist. For instance, participants may have provided inaccurate responses due to social desirability bias, recall bias, or others. To address concerns with subjectivity, we employed a high degree of reflexivity and peer debriefing throughout the research process, as well as a mix of different data sources.

## Conclusion

School-based interventions are important for children’s development in low-income communities, but there are barriers that hinder their effective and continuous implementation. School principals and educators placed their lived experiences at the forefront of the narrative, highlighting the need for participatory approaches to understand the needs of program deliverers and co-design culturally appropriate interventions. The observed limited engagement from educators and caregivers emphasizes the relevance of effective communication strategies that convey the program’s value to foster active involvement and context-specific adoption. A comprehensive approach to school-based interventions comprising health support, adequate training, and regular communication for educators coupled with school-wide actions and community awareness can hold key stakeholders accountable for program ownership, thereby increasing program uptake and long-term maintenance.

### Electronic supplementary material

Below is the link to the electronic supplementary material.

Supplementary Material 1

Supplementary Material 2

## Data Availability

The anonymized transcripts used in the article are freely accessible under: Arnaiz, P. (2023). Transcripts of interviews and focus group interviews pertaining to the perceived acceptance and feasibility of the *KaziKidz* health promotion intervention by educators and caregivers in schools from South Africa [Data set]. Zenodo. 10.5281/zenodo.10033797.

## Abbreviations

CPD: Continued professional development
FGIs: Focus group interviews
LMICs: Low- and middle-income countries
LS/LO: Life skills/life orientation
NCDs: Non-communicable diseases
PA: Physical activity
PE: Physical education
SA: South Africa
