# Supplementary material for: Acceptability and perceived feasibility of the KaziKidz health promotion intervention among educators and caregivers in schools from South Africa: a qualitative descriptive study
Source: BMC Public Health. 2024 Apr 1;24:934. doi: 10.1186/s12889-024-18456-3 (PMC10985953; doi:10.1186/s12889-024-18456-3)
Supplement: Supplementary file 1 — Supplementary Material 1 [file 12889_2024_18456_MOESM1_ESM.docx]

**Additional file 1**

**Interview guides**

**1.1 School principals**

*Start recording*

Introduction

Good morning/afternoon sir/ma’am

Thank you for meeting with us today…

My name is… I am not part of the project; I am here as an external interviewer. My colleague over here is…, who is here to assist with any translation that may be required. In the corner is…, who is just here to scribe and observe the interview.

Today, we are here to talk about the *KaziKidz* program…

*Hand out consent forms*

Start

1. What do you think about the KaziKidz program?

*Prompt: Experiences.*

1. What is the most successful part of KaziKidz?
2. What is the least successful part of the program?
3. How has it affected the wellbeing of children?

*Prompt: Behavioural change, health, requests.*

1. How engaged are the teachers with the program?

*Prompt: Why?*

1. How is your school dealing with the integration of the program into its curriculum?

*Prompt: COVID-19, continuation.*

1. What would you change in general, that you have observed or heard from teachers?

*Prompt: Barriers, suggestions.*

1. How would you adapt it to times of and after COVID-19?
2. Would you recommend the program to other schools? Why yes or why not?

*Prompt: Dissemination to other schools.*

1. What are your wishes for the future with respect to the program?

*Prompt: Continuation, funding.*

End

This was already the last question. But before ending the session, I would like to ask you, if there is anything else that you would like to tell us?

*End recording*

**1.2 Educators**

*Close doors. Ask everyone to take a seat. Request to switch off the phones. Start recording.*

Introduction

Good morning/afternoon everyone

Thank you for meeting with us today…

My name is… I am not part of the project; I am here as an external interviewer. My colleague over here is…, who is here to assist with any translation that may be required. In the corner is…, who is just here to scribe and observe the interview.

Today, we are here to talk about the *KaziKidz* program…

Are there any questions before we begin?

*Hand out consent forms*

Warm Up

Let’s start with brief round of introduction, please introduce yourself to the group…

Let’s move on to the first question:

- - What does health education mean for you?
  - What do you think about how health education is currently taught in the school curriculum?

*Prompts: Is there anything missing (e.g. quality, physical education, nutrition), what do they value?*

Start

Understanding of *KaziKidz*

- 1. Could you explain in your own words what *KaziKidz* is?

*Prompts: Program, structure, goals.*

- 1. What do you think the program *KaziKidz* is trying to achieve?

*Prompts: Health, motor capabilities, social skills.*

Integration of KaziKidz in the school curriculum

- 1. What do you think about *KaziKidz* being introduced in the schools?

*Prompts: Positive changes, unnecessary.*

- 1. How does *KaziKidz* fit into the school curriculum?

*Prompts: Takes time from other subjects, should be leisure time, fits well.*

- 1. What additional value could *KaziKidz* have on top of the current curriculum?

*Prompts: Offers something that the actual curriculum is missing: Quality education, physical education, nutrition, proper development.*

Environment and resources needed for implementation

- 1. How was the participation of teachers in the *KaziKidz* program?

*Prompts: Complaints, motivation.*

- 1. Which resources do schools need to offer *KaziKidz*?

*Prompts: Infrastructure, staff, funding, material, food.*

- 1. What barriers could *KaziKidz* face in the schools?

*Prompts: Staff, infrastructure, funding.*

- 1. How possible is it for children to practice what they have learned outside of school?

*Prompts: At home, communities. Physical activity, good nutrition.*

- 1. How safe is the environment for the children to participate in *KaziKidz*?

*Prompts: Inside and outside of the schools. Excellent, good, average, poor.*

Observations in children and behavioural changes

- 1. How was the participation of children in the *KaziKidz* program?

*Prompts: Exceptionally motivated/active, moderately motivated/active, seldom motivated/active, not motivated/active, indifferent.*

- 1. What changes have you observed in the learners’ behaviour after participation in KaziKidz (and before COVID-19)?

*Prompts: Physical activity, diet, hygiene, request changes in the classroom or school.*

- 1. How has *KaziKidz* influenced children’s health (before COVID-19)?

*Prompts: Weight, feelings (attitudes and emotions).*

- 1. What impact has COVID-19 had on the learners?

*Prompts: Health, behaviours, feelings.*

Personal adoption of and engagement with healthier behaviours

- 1. How do you encourage your learners to be healthier?

*Prompts: Play more, do sports, eat better, cook more, don’t buy fast foods.*

- 1. Since the introduction of *KaziKidz,* have you become more aware of your own health? Please do not consider participation in *KaziHealth*.

*Prompts: How aware are you of your own health risks? Not a lot, a little, neutral, fairly, a lot.*

- 1. What have you done to change your behaviour to be healthier? Everyone can answer.

*Prompts: How motivated are you to change? Physical activity (e.g. joining a gym), nutrition (e.g. home cooking), medical advice (e.g. visit to GP).*

- 1. What were the reasons for changing or not changing?

*Prompts: Barriers (financial, infrastructure, time, support system, own interest).*

Recommendations

- 1. How much would you recommend the *KaziKidz* program to other teachers?

*Prompts: Not a lot, a little, neutral, fairly, a lot.*

- 1. Why would you recommend it or not?

*Prompts: Relevance to learners’ health, material and content covered, ease of access.*

- 1. What are some suggestions that you would like the *KaziKidz* to improve on?

*Prompts: Additional aspects and/or focus areas, material and/or resources, functionality, relevance and accessibility.*

- 1. How can *KaziKidz* be implemented in the schools in times of and after COVID-19?

*Prompts: Adaptations, feasibility.*

End

This was already the last question. But before ending the session, I would like to ask you, if there is anything else that you would like to tell us?

*End recording*

**1.3 Caregivers**

*Close doors. Ask everyone to take a seat. Request to switch off the phones. Start recording.*

Introduction

Good morning/afternoon everyone

Thank you for meeting with us today…

My name is… I am not part of the project; I am here as an external interviewer. My colleague over here is…, who is here to assist with any translation that may be required. In the corner is…, who is just here to scribe and observe the interview.

Today, we are here to talk about the *KaziKidz* program…

Are there any questions before we begin?

*Hand out consent forms*

Warm Up

Let’s start with brief round of introduction, please introduce yourself to the group…

Let’s move on to the first question:

- What does it mean for you to be healthy?
- How heathy is your family?

Start

Understanding of the health promotion intervention *KaziKidz*

1. What do you think the program *KaziKidz* is trying to achieve?

*Prompts: Health, movement, happiness.*

Integration of *KaziKidz* in the school curriculum

1. What do you think about the current school curriculum?

*Prompts: Is there anything missing (e.g. quality, physical education, nutrition), what do they value.*

1. What do you think about KaziKidz being introduced in the schools?

*Prompts: Positive changes, unnecessary.*

Environment and resources needed for implementation

1. Which resources do schools need to offer KaziKidz?

*Prompts: Playground and sport area, availability of fresh food.*

1. How possible is for children to practice what they have learned outside of school?

*Prompts: RESOURCES at home, communities. Physical activity, good nutrition.*

1. How safe is the environment for the children to participate in KaziKidz?

*Prompts: Inside and outside of the schools. Excellent, good, average, poor.*

Observations in children and behavioural changes

1. How did your children like the program?

*Prompts: Participation, motivation, talked about it at home, indifferent.*

1. What changes have you observed in your child’s behaviour after participation in KaziKidz?

*Prompts: Physical activity, diet, hygiene, request changes at home*

1. How has *KaziKidz* influenced your child’s health?

*Prompts: Weight, feelings (attitudes and emotions).*

Personal adoption of and engagement with healthier behaviours

1. How do you encourage your child to be healthier?

*Prompts: Play more, do sports, eat better, cook more, don’t buy fast foods*

1. Since the introduction of *KaziKidz,* have you become more aware of your own health?

*Prompts: How aware are you of your own health risks? Not a lot, a little, neutral, fairly, a lot*

1. What have YOU done to change your behaviour to be healthier?

*Prompts: How motivated are you to change? Physical activity (e.g. joining a gym), nutrition (e.g. home cooking), medical advice (e.g. visit to GP).*

1. What were the reasons for changing or not changing?

*Prompts: BARRIERS (financial, infrastructure, time, support system, own interest).*

1. How has COVID-19 influenced your family’s health?

*Prompts: B*ehaviours*, feelings*

Recommendations

1. How would you recommend the *KaziKidz* program to other parents?

*Prompts: not a lot, a little, neutral, fairly, a lot.*

1. Why would you recommend it or not?

*Prompts: Relevance to child’s or own health, access to materials, relevancy.*

1. What are some suggestions that you would like the *KaziKidz* program to improve on?

*Prompts: Additional areas, material and resources, relevance, and accessibility (digital).*

End

This was already the last question. But before ending the session, I would like to ask you, if there is anything else that you would like to tell us?

*End recording*
