# Supplementary material for: Acceptability and perceived feasibility of the KaziKidz health promotion intervention among educators and caregivers in schools from South Africa: a qualitative descriptive study
Source: BMC Public Health. 2024 Apr 1;24:934. doi: 10.1186/s12889-024-18456-3 (PMC10985953; doi:10.1186/s12889-024-18456-3)
Supplement: Supplementary file 2 — Supplementary Material 2 [file 12889_2024_18456_MOESM2_ESM.docx]

**Additional file 2**

| **A 15-point checklist of criteria for good thematic analysis^a^** | | |
| --- | --- | --- |
| **Process** | **No.** | **Criteria** |
| Transcription | 1 | The data have been transcribed to an appropriate level of detail, and the transcripts have been checked against the tapes for ‘accuracy’. |
| Coding | 2 | Each data item has been given equal attention in the coding process. |
|  | 3 | Themes have not been generated from a few vivid examples (an anecdotal approach), but instead the coding process has been thorough, inclusive and comprehensive. |
|  | 4 | All relevant extracts for all each theme have been collated. |
|  | 5 | Themes have been checked against each other and back to the original data set. |
|  | 6 | Themes are internally coherent, consistent, and distinctive. |
| Analysis | 7 | Data have been analyzed - interpreted, made sense of - rather than just paraphrased or described. |
|  | 8 | Analysis and data match each other - the extracts illustrate the analytic claims. |
|  | 9 | Analysis tells a convincing and well-organized story about the data and topic. |
|  | 10 | A good balance between analytic narrative and illustrative extracts is provided. |
| Overall | 11 | Enough time has been allocated to complete all phases of the analysis adequately, without rushing a phase or giving it a once-over-lightly. |
| Written report | 12 | The assumptions about, and specific approach to, thematic analysis are clearly explicated. |
|  | 13 | There is a good fit between what you claim you do, and what you show you have done – i.e., described method and reported analysis are consistent. |
|  | 14 | The language and concepts used in the report are consistent with the epistemological position of the analysis. |
|  | 15 | The researcher is positioned as active in the research process; themes do not just ‘emerge’. |

^a^Braun V, Clarke V. Using Thematic Analysis in Psychology. Qualitative Research in Psychology (2006) 3(2):77-101. doi: 10.1191/1478088706qp063oa.
